# Information processing and synaptic plasticity at hippocampal mossy fiber terminals

**DOI:** 10.3389/fncel.2014.00028

**Published:** 2014-02-04

**Authors:** Alesya Evstratova, Katalin Tóth

**Affiliations:** Faculty of Medicine, Department of Psychiatry and Neuroscience, Quebec Mental Health Institute, Université LavalQuebec City, QC, Canada

**Keywords:** mossy fiber, synaptic plasticity, CA3 region, hippocampal, interneurons, information transfer

## Abstract

Granule cells of the dentate gyrus receive cortical information and they transform and transmit this code to the CA3 area via their axons, the mossy fibers (MFs). Structural and functional complexity of this network has been extensively studied at various organizational levels. This review is focused on the anatomical and physiological properties of the MF system. We will discuss the mechanism by which dentate granule cells process signals from single action potentials (APs), short bursts and longer stimuli. Various parameters of synaptic interactions at different target cells such as quantal transmission, short- and long-term plasticity (LTP) will be summarized. Different types of synaptic contacts formed by MFs have unique sets of rules for information processing during different rates of granule cell activity. We will investigate the complex interactions between key determinants of information transfer between the dentate gyrus and the CA3 area of the hippocampus.

## Introduction

One of the most studied synapses in the central nervous system (CNS) is the dentate mossy fiber (MF) input onto hippocampal CA3 pyramidal cells. Nevertheless, in spite of the large amount of information about the organization and function of this synapse, we are far from the complete understanding of this complex structure. MFs play a unique role in the transformation of incoming cortical signals and ensure the faithful transfer of the resulting code to the CA3 area. The dentate gyrus is the first relay in the cortico-hippocampal loop, it is involved in the translation of densely coded cortical signals to sparse and specific hippocampal code, which is essential for hippocampal memory formation (Acsady and Kali, 2007). The high-pass filter nature of the dentate–CA3 circuit allows the conversion of multiple place fields of dentate place cells into a single receptive field observed in CA3 place cells. This process critically relies on the exact firing pattern of dentate granule cells and results in more orthogonalized stimulus representation in the CA3 area (Leutgeb et al., 2007). Likewise, in case of repetitive stimulus presentation or autonomous memory trace replay, presynaptic MF long-term plasticity (LTP) may help the orthogonalization process, creating an opportunity for the faithful re-activation of the same cornu ammonis region 3 (CA3) circuit pattern.

The aim of this review is to provide an overview about the cellular processes responsible for the transformation of entorhinal inputs into hippocampal codes by granule cells of the dentate gyrus. We will review how single granule cell action potential (AP) influence postsynaptic CA3 pyramidal cells and interneurons, we will discuss the unique morphological and physiological properties of the presynaptic specializations of these cells and how these features contribute to postsynaptic signaling. Next, we will discuss how communication between the dentate gyrus and the CA3 area is influenced by changes in presynaptic firing frequencies. Finally, the complex and diverse synaptic plastic properties of MF synapses will be summarized and discussed.

## Structural properties of hippocampal mossy fibers

MFs are nonmyelinated axons of granule cells, located in the dentate gyrus. These axons are projecting primarily to the proximal parts of CA3 pyramidal cell dendrites and distal dendrites of interneurons (Acsady et al., 1998). Axonal tracing revealed that during its passage through the hilus, each MF gives rise to a number of branching collaterals, contacting various hilar neurons, while the main axons continues toward the pyramidal cell layer of the hippocampal CA3 area. MFs do not form collaterals within the CA3 area, they are organized in a laminar fashion along the pyramidal cell layer, their projection is restricted to the stratum lucidum.

**Figure 1:**
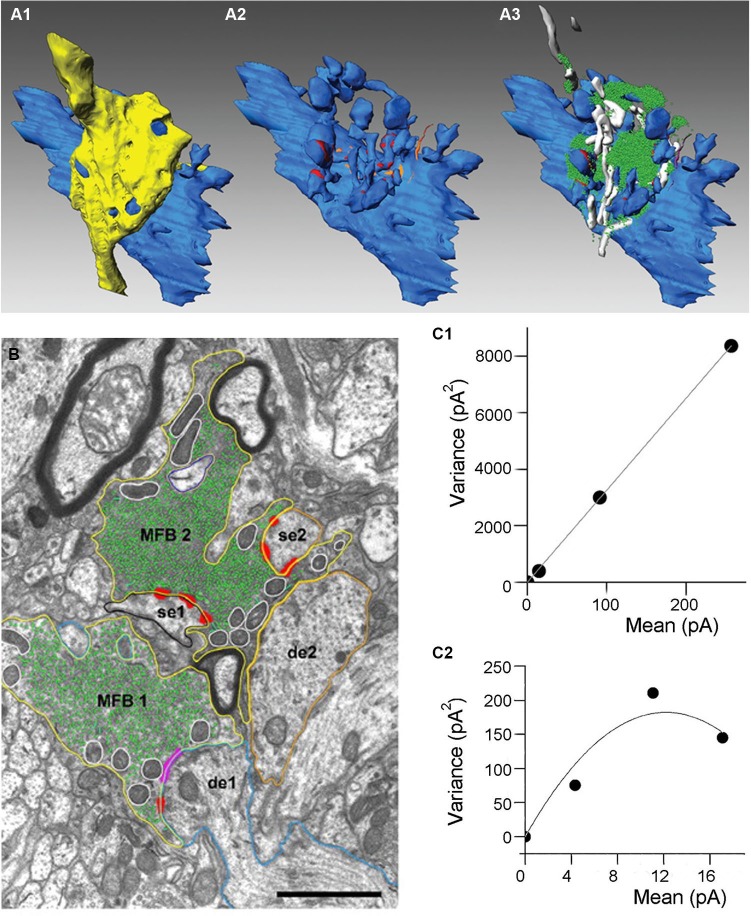
**Anatomical organization and quantal parameters of MF synapses**. **(A)** Three-dimensional reconstructions of an adult MF bouton and its postsynaptic target dendrite. **(A1)** Volume reconstructions of bouton (yellow) and its postsynaptic target dendrite (blue). **(A2)** Distribution of the two membrane specializations, active zones (in red) and puncta adherentia (in orange) on the postsynaptic target dendrite. Note, that active zones were mainly located on the spiny excrescences, whereas puncta adherentia were exclusively found at the dendritic shaft. **(A3)** Organization of the pool of synaptic vesicles (green dots) and mitochondria (in white) at an individual MF bouton. Adapted from Rollenhagen and Lübke (2010). **(B)** Electron microscopic image of two adjacent MF boutons (MFB1, MFB2). Both boutons outlined in yellow terminate on different dendritic segments (de1, blue contour; de2, orange contour), but preferentially on spiny excrescences (se1, black contour; se2, orange contour). Active zones (red), puncta adherentia (magenta), mitochondria (white contours), and individual synaptic vesicles (green). Scale bar corresponds to 1 μm, adapted from Rollenhagen and Lübke (2007). **(C)** Variance-mean analysis of mossy fiber-CA3 pyramidal cell **(C1)** and mossy fiber-interneurone **(C2)** synapses. Data obtained at three different calcium conditions (1 mM, 2.5 mM and 3.8 mM of CaCl_2_). Solid lines correspond to either liner **(C1)** or parabolic fit **(C2)**. Adapted from Lawrence and McBain (2003).

Each MF forms several, sparsely located large boutons (3–10 μm diameter), which envelop postsynaptic thorny excrescences emerging from CA3 pyramidal cell apical dendrites, while 2–4 tiny filopodial extensions stemming from these large boutons innervate dendrites of inhibitory interneurons (Figure 1A). In average, one MF fiber makes four different types of excitatory synapses: 7–12 large terminals contacting hilar mossy cells; 11–18 large terminals innervating CA3 pyramidal cells; 120–150 small terminals forming synapses on hilar interneurons and 40–50 filopodial extensions terminating on CA3 interneurons (Acsady et al., 1998). MF boutons contact multiple dendritic segments of the same or different pyramidal neurons (Chicurel and Harris, 1992; Galimberti et al., 2006). The number of granule cells converging on CA3 pyramidal cell is remarkably high, an apical dendrite of a single pyramidal cell is contacted by ~50 MF boutons each originating from different granule cells (Amaral et al., 1990).

Three different types of vesicles have been described within the giant MF boutons: majority of them are small clear vesicles (~40 nm) containing glutamate, there are also large dense-core vesicles filled with various neuropeptides (dynorphin, enkephalin, cholecystokinin, neuropeptide Y and neurokinin-β) and large clear vesicles (~200 nm). MF boutons also contain the neuromodulator ATP/adenosine and Zn^2+^, which is co-localised in the same vesicles as glutamate. Large MF boutons forming excitatory inputs on CA3 pyramidal cells exhibit several unique properties shaping neurotransmission at these synapses. MF boutons have multiple release sites, which were initially observed using electron microscopy of reconstructed terminals (Chicurel and Harris, 1992; Rollenhagen et al., 2007; Rollenhagen and Lubke, 2010). According to these reconstructions the number of release sites varies between 18–45 with a mean surface area of 0.1 μm^2^ and a short distance of 0.45 μm between individual sites, Figure 1B (Rollenhagen et al., 2007). The short distance between release sites indicates that crosstalk may occur either presynaptically via Ca^2+^ diffusion or postsynaptically via glutamate spillover from neighboring release sites. MF terminals contain very large number of synaptic vesicles, ~25,000 vesicles are observed in young rats, approximately 75% of these vesicles corresponds to the reserve pool (Rollenhagen et al., 2007). The putative releasable pool of ~5700 vesicles was estimated from 3D-reconstructions of MF boutons (Rollenhagen et al., 2007). In contrast, direct capacitance measurement from MF boutons suggests that ~1400 vesicles comprise the readily releasable pool (RRP; Hallermann et al., 2003). Moreover, the number of vesicles at individual release sites is also very high, which might be important for the support of reliable neurotransmission during high-frequency activity.

## Neurotransmitter release triggered by single action potentials

Synaptic transmission is primarily based on the regulated release of neurotransmitter from synaptic vesicles. When an AP arrives at the presynaptic axonal terminal, depolarization opens voltage-gated Ca^2+^ channels (VGCCs) and Ca^2+^ influx triggers the fusion of synaptic vesicles docked and primed at the active zone of the presynaptic plasma membrane. Subsequently, neurotransmitter is released into the synaptic cleft and diffuses to the postsynaptic membrane to activate neurotransmitter receptors. Finally, synaptic vesicles are retrieved via endocytosis in order to restore the releasable vesicle pool (Littleton, 2006; Ryan, 2006). Thus, synaptic transmission depends not only on the number of active zones and vesicles available for release, but also on the properties of the release machinery and composition of pre and postsynaptic receptors and signaling cascades.

### Mossy fiber boutons

An important parameter of neurotransmission is the quantal size (Q), the amplitude of a synaptic response evoked by glutamate release from a single vesicle. This parameter characterizes the amount of glutamate packed into the vesicle and was calculated for this synaptic connection using stationary variance-mean analysis at different Ca^2+^ concentrations, Q = 29 pA, Figure 1D (Lawrence et al., 2004). MF-pyramidal cell synapses exhibit very low release probability (*p* = 0.2–0.28), (von Kitzing et al., 1994), but due to the large number of release sites unitary excitatory postsynaptic currents (EPSCs) and excitatory postsynaptic potentials (EPSPs) can reach unusually high amplitude (1 nA and 10 mV, respectively) (Bischofberger et al., 2006).

The properties of APs recorded from boutons are different from the APs observed in the soma of granule cells. In particular, they are almost twice faster (half-duration 379 ± 8 μs compared to 678 ± 45 μs at the some) and have less pronounced afterdepolarization (Geiger and Jonas, 2000). These properties are mediated by the dendrotoxin (DTX)-sensitive K^+^ channels (presumably composed of Kv1.1α/1.4α or Kv1.1α/β subunits; Coghlan et al., 2001). The combination of low activation and deactivation thresholds (~60 mV) provides rapid and complete reset of the membrane potential after AP generation (Geiger and Jonas, 2000). While the properties of K^+^ channels shape the repolarizating phase of AP, its rising phase depends mainly on the activation of Na^+^ channels. In contrast to many other axonal terminals, MF boutons have active properties and express a large number of Na^+^ channels (~2000 per bouton; Engel and Jonas, 2005). Moreover, these channels have faster inactivation kinetic and serve to boost presynaptic APs and enhance calcium influx, rather than to ensure AP propagation.

Interestingly, not only APs, but also subthreshold dendritic synaptic inputs participate in information processing at this synapse. Excitatory presynaptic potentials evoked by such inputs can propagate along the granule cell axon and modulate glutamate release evoked by APs (Alle and Geiger, 2006). A single presynaptic subthreshold potential combined with APs resulted in a significantly larger postsynaptic response; this effect was dependent on calcium signaling and direct voltage modulation of vesicle release.

Presynaptic calcium currents at MF boutons are primarily mediated by P/Q-type Ca^2+^ channels (Castillo et al., 1994; Breustedt et al., 2003; Miyazaki et al., 2005). Whole-bouton recordings were used to investigate directly the kinetic properties of these channels. MF boutons have large high-voltage-activated calcium currents, with fast activation and deactivation (Bischofberger et al., 2002). Furthermore, computational modeling of these calcium currents indicated that the gating kinetics of the Ca^2+^ channels and sharp, pulse-like shape of the presynaptic AP act together to maximize calcium influx. The presence of Na^+^ channels in the bouton leads to additional ~40 mV increase in the depolarization and therefore can amplify calcium currents by two fold (Engel and Jonas, 2005). Based on the large amplitude of Ca^2+^ currents evoked by single APs (Bischofberger et al., 2002) and assuming a single channel current of ~0.2 pA (Gollasch et al., 1992; Brandt et al., 2005), it has been proposed that approximately 850 Ca^2+^ channels contribute to the peak amplitude of calcium signals, which corresponds to ~23 opened Ca^2+^ channels per release site.

Glutamate released from MF boutons mediates fast ionotropic responses at the postsynaptic membrane mainly by activating α-amino-3-hydroxy-5-methyl-4- isoxazolepropionic acid (AMPA)-type receptors (Lanthorn et al., 1984; Neuman et al., 1988; Ito and Sugiyama, 1991; Jonas et al., 1993). Ca^2+^-impermeable AMPARs prevail at mature MF-CA3 pyramidal neuron synapses, while Ca^2+^-permeable AMPARs undergo developmental regulation (Ho et al., 2007). In contrast to other hippocampal synapses, thorny excrescences of CA3 pyramidal neurons express low density of N-Methyl-D-aspartic acid receptors (NMDARs), which nevertheless provide a small, but measurable, NMDAR-mediated current upon glutamate release from MFs (Jonas et al., 1993; Weisskopf and Nicoll, 1995).

### Mossy fiber filopodial extensions

In contrast to large boutons, tiny filopodial extensions selectively contacting CA3 interneurons have only 1–2 release sites, which was initially observed using electron microscopy (Acsady et al., 1998) and later confirmed using several electrophysiological approaches, including variance-mean analysis (Lawrence et al., 2004). From the same set of experiments quantal size was estimated to be ~27 pA, which is similar to the quantal size at synapse between large MF boutons and CA3 pyramidal cells (Figure 1C). Interestingly, the probability of release at filopodial extensions varied between 0.34 and 0.51 during normal calcium concentration (2.5 mM) and increased up to 0.44–0.78 at high calcium concentration (3.8 mM), which is several folds higher than at MF-CA3 pyramidal cell synapses (Lawrence et al., 2004). The high probability of release could be explained by active properties of filopodial extensions, where activation of Na^+^ channels may lead to more prominent increase in local AP amplitude than in the main bouton (Engel and Jonas, 2005).

The total number of vesicles within filopodial extensions is two orders of magnitude smaller than in the large boutons (200–700 vesicles; Rollenhagen et al., 2007). The number of releasable vesicle has not been quantitatively assessed in filopodia. However, given that size of the RRP at large mossy boutons is ~5% of the total number of vesicles, the putative RRP at filopodial extensions could contain 10–40 vesicles.

The filopodial extensions are too small for direct electrophysiological recordings; however it’s possible to fill individual MF terminals with a membrane-permeable calcium indicator and perform simultaneous calcium imaging at the large bouton and the filopodia (Pelkey et al., 2006). Similarly to the main bouton, calcium currents in small filopodia are primarily mediated by the activation of P/Q-type Ca^2+^ channels, with small contribution from N-type channels (Pelkey et al., 2006). In spite of this similarity, different signaling cascades control the activation of Ca^2+^ channels during repetitive stimulation, this mechanism will be described in more details below.

CA3 interneurons located in the stratum lucidum receive synapses from MFs or local CA3 pyramidal cell collaterals. MF inputs could activate either synapses containing postsynaptic calcium-permeable AMPARs sensitive to philanthotoxin (Toth and Mcbain, 1998) or synapses expressing calcium-impermeable AMPARs (Toth et al., 2000), while inputs from CA3 pyramidal cells only contact synapses expressing only calcium-impermeable AMPARs. Biocytin filling and subsequent visualization of recorded interneurons did not reveal any preferential localization of calcium-permeable and -impermeable AMPARs on specific subtypes of the stratum lucidum interneurons (Lei and Mcbain, 2002). However, an important correlation between calcium permeability of AMPA receptors and the presence of NMDA receptors in a given synapse was unveiled. At MF-interneuron synapses where AMPARs are calcium permeable only a small fraction of evoked EPSPs are mediated by NMDARs (Lei and Mcbain, 2002). Therefore, inhibition provided onto CA3 pyramidal cells is shaped largely by fast AMPAR currents which controls the precise timing of APs. In contrast, MF-interneuron synapses containing calcium-impermeable AMPARs exhibit a prominent NMDAR component. Here, activation of NMDARs leads to substantially slower EPSP decays and thus a much longer time window for synaptic integration (Maccaferri and Dingledine, 2002), this, in turn, can be translated to a wider temporal window for spike integration in CA3 pyramidal cells. Thus, two different modes of inhibition could be triggered by the activation of MF inputs at synapses composed of distinct sets of postsynaptic glutamate receptors.

## Information processing during burst firing

The average firing rate of granule cells recorded *in vivo* is very low, approximately 0.01–0.1 Hz (Jung and Mcnaughton, 1993). However, spiking activity increase considerably when granule cells participate in information transfer. For instance, the firing frequency of dentate gyrus granule cells can increase up to 10–50 Hz during place cell activity, with the individual bursts reaching 100–300 Hz (Jung and Mcnaughton, 1993; Skaggs et al., 1996; Gundlfinger et al., 2010). These bursts participate in spatial information coding. Therefore, the mechanism by which granule cell burst firing is transmitted at the MF synapses is a key element of information transfer from the dentate gyrus to the CA3 area of the hippocampus. Short-term plasticity plays a significant role in this process, it is primarily expressed presynaptically and it plays a substantial role in the regulation of the balance between excitation and inhibition and the resultant network activity.

### Mossy fiber boutons

**Figure 2:**
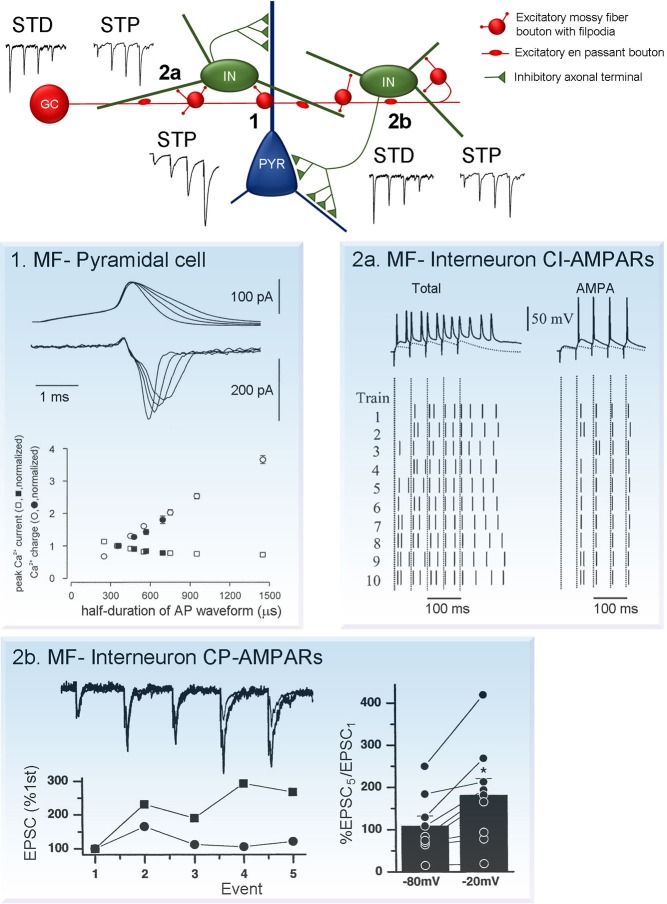
**Short-term plasticity at synapses formed by MF boutons on CA3 neurons. (Center)** Schematic diagram showing connection formed by individual granule cells with CA3 pyramidal cells and interneurons. Note that the number of contacts with interneurons is large, compared to pyramidal cells. Moreover, each interneuron forms multiple inhibitory contacts on pyramidal cells. This network organization leads to the prevalence of inhibition on CA3 pyramidal cells during low granule cell activity. However, during granule cell burst firing MF synapses on pyramidal cells undergo robust facilitation short-term potentiation (STP, synapse **1**), while synapses on interneurons either depress or exhibit mild facilitation short-term depression (STD/STP, synapses **2a** and **2b**). As a result, MF neurotransmission leads to reliable AP generation at postsynaptic CA3 pyramidal neurons. Plasticity traces adapted from Toth and Mcbain (2000). Examples of mechanism underlying short-term plasticity at each type of the synapses are shown on panels **1**, **2a** and **2b**. **(1)** MF-pyramidal cell synapses: AP broadening increases presynaptic calcium influx. Top (upper traces), current-clamp recordings of APs and top (lower traces), corresponding calcium currents. Bottom, peak current amplitude (squares) and integral (circles; determined in a 5 ms time window), plotted against half-duration of the voltage-clamp command. Open symbols indicate values for mock APs, filled symbols represent realistic APs. Adapted from Geiger and Jonas (2000). **(2a)** MF-interneuron synapses expressing calcium-impermeable AMPARs (CI-AMPARs): contributions of NMDARs and AMPARs to AP transmission. EPSP/AP sequences elicited by trains of stimuli (5 stimuli, 20 Hz) in the absence (left column) or presence (right column) of (2R)-amino-5-phosphonovaleric acid (APV). Upper trace shows a single representative example of response evoked by stimulation. Lower panel shows the raster plot of spikes induced by each of the 5 stimuli in the 10 trials. Each short vertical line represents a single AP, dashed lines represent the timing of the stimulus artifact. Adapted from Lei and Mcbain (2002). **(2b)** MF-interneuron synapses expressing calcium-permeable AMPARs (CP-AMPARs): voltage-dependent relief from polyamine block enhances facilitation at depolarized potentials. Top, trains of five MF EPSCs evoked by 20 Hz stimulation at two holding potentials (−80 and −20 mV). Bottom, normalizing the first EPSCs in the trains reveals a greater degree of facilitation at −20 mV. Right, Summary histogram shows that the EPSC_5_/EPSC_1_ ratio always larger at −20 mV, polyamine block is relieved. (Toth and Mcbain, 2000).

One of the remarkable properties of the MF-CA3 pyramidal cell synapse is their unusually high degree of facilitation during repetitive stimulation (Figure 2). For example, a 15 stimuli train delivered at 4 Hz can increase EPSC amplitude more than 10 times (Toth et al., 2000). Moreover, natural stimulation patterns including place cell burst firing, can efficiently lead to short term facilitation increasing postsynaptic responses by 400–500% (Gundlfinger et al., 2010). The large degree of facilitation is associated with low initial release probability, which is significantly augmented during repetitive stimulation. Short-term facilitation is supported by a large RRP providing constant and reliable supply of vesicles during long high-frequency stimuli.

The exact molecular mechanism of synaptic facilitation at MF-CA3 pyramidal cell synapses have been extensively studied, while several key components are well defined, some aspects remain controversial. One of the key players in short-term facilitation is the prominent increase in intra-bouton calcium concentration during repeated stimuli. This, in turn, is directly linked to enhanced glutamate release due to calcium-dependent regulation of synaptic vesicle exocytosis. Fast APs recorded in MF boutons undergo activity-dependent broadening during repetitive stimulation which results in substantially increased presynaptic calcium influx, Figure 2. (Geiger and Jonas, 2000). Interestingly, although AP broadening decreases the peak amplitude of calcium currents, it increases the total calcium influx per spike and leads to augmented glutamate release. This suggests that integral calcium current, rather than its peak amplitude determines release probability.

Application of the membrane permeable, slow calcium chelator ethylene glycol tetraacetic acid, tetra (acetoxymethyl ester) (EGTA-AM) significantly reduces the degree of facilitation (Regehr et al., 1994; Salin et al., 1996; Tzounopoulos et al., 1998), indicating that accumulation of residual calcium during stimulation trains is essential. Calcium release from ryanodine sensitive stores might also be involved in short-term facilitation, as the blockade of calcium release from stores with ryanodine significantly reduces paired pulse facilitation (Scott and Rusakov, 2006; Scott et al., 2008). Interestingly, in other systems, ryanodine failed to have similar effect on synaptic facilitation (Carter et al., 2002; Shimizu et al., 2008). It is yet to be determined whether calcium release from stores plays a significant role in synaptic signal integration during longer, natural-like stimulations patterns.

Presynaptic glutamate receptors can play a crucial role in short-term facilitation as their activation can alter the amplitude of subsequent postsynaptic signals. Activation of presynaptic metabotropic glutamate receptors (mGluRs) leads to the suppression of glutamate release most likely via the inhibition of VGCCs (Castillo et al., 1994; Min et al., 1998; Kamiya and Ozawa, 1999; Toth et al., 2000; Pelkey et al., 2006). Endogenous glutamate can also activate presynaptic kainate receptors (KARs), leading to the enhancement of AP evoked calcium influx in the terminal, and therefore increased facilitation (Lauri et al., 2001; Schmitz et al., 2001; Scott et al., 2008; Dargan and Amici, 2009). Interestingly, there are also data suggesting that during short stimulation bursts (0.5–25 Hz) depression of glutamate release induced via mGluRs predominantly shapes the evoked responses while the effect of KARs activation is very small (Kwon and Castillo, 2008b). These findings indicate that glutamate release from MFs during repetitive stimulation acts not only postsynaptically, but also activates several types of presynaptic autoreceptors triggering distinct molecular cascades which modulate calcium signaling and neurotransmitter release.

At the postsynaptic site glutamate receptor activation can also undergo short-term changes during bursts. In particular, activation of postsynaptic KARs can generate slow depolarizing synaptic currents, which contribute minimally to postsynaptic potentials during low-frequency stimulation (Castillo et al., 1997; Vignes and Collingridge, 1997). However, these currents start to play a significant role in postsynaptic AP generation during repetitive high-frequency MF firing (Kwon and Castillo, 2008b; Sachidhanandam et al., 2009) and express short-term depression (STD) mediated by NMDARs (Rebola et al., 2007).

### Mossy fiber filopodial extensions

MF synapses established between filopodial extensions and interneurons can exhibit both short-term potentiation (STP) and depression during repetitive stimulation, Figure 2 (Toth et al., 2000; McBain, 2008). Interestingly, the direction of plasticity is independent of the presence of calcium-permeable AMPARs and it is rather determined by the initial release probability. In general, potentiation at MF-interneuron synapses is remarkably small compared to that observed at synapses terminating on pyramidal cells (4X increase vs. 10X increase, at 4 Hz). Moreover, while at synapses terminating pyramidal cells significant facilitation could be observed as low as 0.1 Hz stimulation frequency, at synapses impinging on interneurons facilitation only occurs at stimulation frequencies higher than 1–2 Hz (Toth et al., 2000). The magnitude of synaptic response evoked by MF inputs in interneurons and pyramidal cells largely depends on the frequency of granule cell firing. In fact, it’s believed that short-term plasticity at both types of synapses is crucial for hippocampal information processing and regulates network activity.

A unique form of short-term plasticity exists at synapses expressing calcium-permeable AMPARs. In contrast to conventional forms of short-term plasticity it is induced postsynaptically. Calcium-permeable AMPARs under resting conditions are tonically blocked with intracellular ployamines, such as spermine and spermidine (Kamboj et al., 1995; Koh et al., 1995), this blockade is use-and voltage-dependent and requires multiple receptor activations (i.e., repetitive stimulation) to remove polyamine from the channel pore (Bowie and Mayer, 1995). Thus, due to the use-dependence of polyamine block, current flowing through calcium-permeable AMPARs increases during presynaptic bursts. However, as polyamine block and the resulting facilitation are voltage-dependent this form of STP is almost absent at resting membrane potentials and becomes prominent only when neurons are depolarized (Toth et al., 2000). Due to these unique properties, under physiological conditions, calcium-permeable AMPARs may play a role of coincidence detectors.

Synapses expressing calcium-impermeable AMPARs also contain N-methyl D-aspartate receptor subtype 2B (NR2B)-lacking NMDARs. The NMDA component significantly influences temporal summation and increases the number of evoked postsynaptic APs, Figure 2a (Lei and Mcbain, 2002). In contrast, synapses with calcium-permeable AMPARs co-express NR2B-containing NMDARs (Bischofberger et al., 2002; Lei and Mcbain, 2002). At this type of synapse repetitive stimulation results in rapid and brief EPSPs and APs with little jitter, Figure 2b (Lei and Mcbain, 2002; Walker et al., 2002). Thus, these two types of MF synapses are designed for two different modes of neurotransmission: calcium permeable AMPAR expressing synapses for precise and rapid synaptic transmission; synapses containing calcium-impermeable APMARs for large depolarization and multiple APs without accurate timing (Lawrence and McBain, 2003; Jonas et al., 2004).

Similarly to MF-CA3 pyramidal cell synapses, activation of presynaptic mGluRs during repetitive stimulation decreases the amplitude of facilitation at synapses terminating on interneurons (Toth et al., 2000; Cosgrove et al., 2011), however this effect is less pronounced (Kamiya and Ozawa, 1999). In contrast, the enhancement of synaptic transmission by kainate autoreceptors is specific to MF-pyramidal cell synapses. Inhibition of KARs does not alter synaptic currents and presynaptic calcium influx at synapse formed onto inhibitory interneurons (Scott et al., 2008).

### Activity-dependent network output

The combined effect of the aforementioned diverse postsynaptic responses observed in MF targets in response to presynaptic bursts will largely depend on the particular pattern of the stimuli. Frequency-dependent alteration of direct excitatory and indirect feed-forward inhibitory responses will determine network output. This combined output is influenced by the connectivity pattern of MFs and its targets and the temporal summation of synaptic inputs.

Anatomical data indicate that MFs form larger number of synaptic contacts on interneurons than on CA3 pyramidal cells. Since inhibitory neurons receiving MF inputs contact several hundreds of CA3 pyramidal cells, granule cell firing also triggers powerful disynaptic feed-forward inhibition. While MF-principal cell synapses have low release probability, they also exhibit very large facilitation during repetitive stimulation. In contrast, similar stimulation at the MF-interneuron synapse leads to synaptic depression or mild potentiation.

The balance between excitation and inhibition is a key determinant of network output and it is developmentally regulated. In young animals, polysynaptic feed-forward inhibition of CA3 pyramidal cells facilitates during burst activity, while in adults both facilitation and depression can occur (Torborg et al., 2010). The temporal precision of postsynaptic APs is not influenced by feed-forward inhibition, but it is rather determined by the amplitude and kinetic properties of excitatory inputs. However, feed-forward inhibition plays a key role in the regulation of CA3 pyramidal cell excitability and prevents the development of excitatory plateaus and repetitive postsynaptic cell firing. In adults, a shift between the facilitation and depression of network inhibition may act as a switch between tonic and burst firing modes (Torborg et al., 2010).

Frequency-dependent facilitation of MF neurotransmission has been demonstrated *in vivo*, using recordings from monosynaptically connected granule cell-pyramidal cells (Henze et al., 2002). The probability of postsynaptic AP firing rose rapidly with increased granule cell firing frequency. Four-five presynaptic APs were sufficient to reach maximum AP probability in CA3 pyramidal neurons. In contrast, at granule cell–interneuron synapses, increase in the number of the presynaptic spikes delivered at 100 Hz did not enhance the probability of postsynaptic APs. These data indicate that various forms of short-term plasticity at different postsynaptic targets lead to frequency-dependent alterations in the net postsynaptic response.

Anatomical data demonstrating that the number of inhibitory postsynaptic targets of MFs is several times bigger than the number of innervated pyramidal cells (Acsady et al., 1998), and physiological data depicting the mechanism by which the balance between excitation and inhibition can be shifted when granule cell firing rate is altered (Henze et al., 2002) suggest that the combined effect of granule cell activity in a behaving animal is inhibitory rather than excitatory. This idea is supported by *in vivo* data demonstrating that during cortical UP states activity in entorhinal cortex, dentate gyrus and most CA1 neurons increased, in contrast neurons in the CA3 are not active (Isomura et al., 2006). Similarly, dentate spikes resulting from the synchronous excitation of dentate granule cells by entorhinal stellate cells, decreased multiunit activity recorded in the CA3 area (Bragin et al., 1995). The general inhibitory effect of granule cell activity is also supported by the simultaneous and opposite effects of sensory stimulation on population activity in the CA1 area and the dentate gyrus; perforant pathway responses in granule cells are facilitated by sensory stimulation while CA1 population spikes are reduced (Herreras et al., 1988).

## Synaptic plasticity

Synaptic efficacy at MF synapses, similarly to other synapses in the CNS, can persistently be altered. Remarkably, synapses formed by MFs on both pyramidal cells and interneurons show unusual forms of LTP, and almost all elements including the direction of plasticity, induction and expression sites manifest in a target cell-dependent manner.

### Long-term plasticity at mossy fiber-pyramidal cell synapses

LTP of MF inputs on CA3 pyramidal neurons can be evoked by various high frequency stimulation protocols (Yamamoto et al., 1980; Zalutsky and Nicoll, 1990; Castillo et al., 1994; Nicoll and Schmitz, 2005), and with natural granule cell firing patterns (Mistry et al., 2011). Interestingly, only firing patterns containing high frequency bursts with low average firing rate were efficient to induce LTP, such patterns are characteristic of granule cell firing during memory tasks (Mistry et al., 2011).

**Figure 3:**
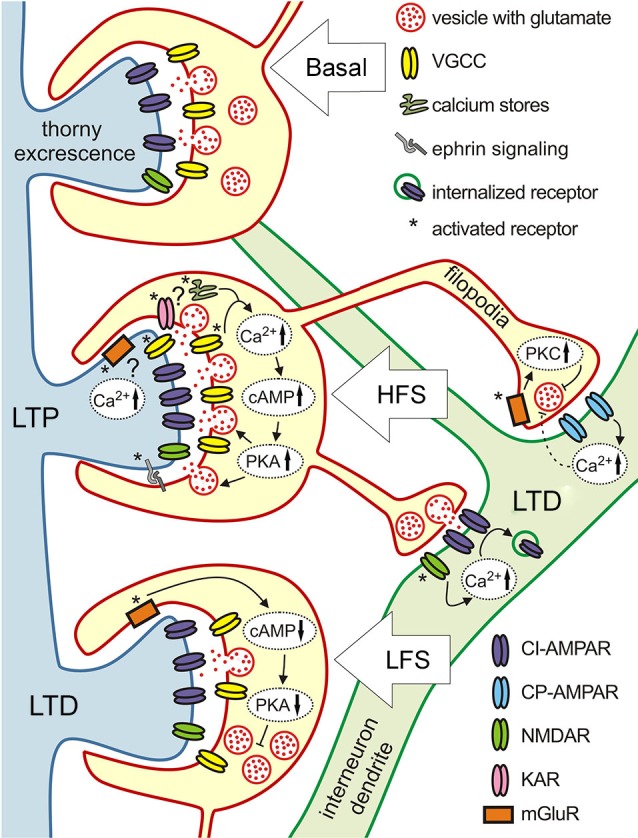
**Target-specific plasticity at three different types of MF synapses**. Schematics illustration of the various molecular mechanisms underlying different forms of plasticity at MF synapses terminating on pyramidal cells (blue dendrite with thorny excrescencies) and inhibitory interneurons (green dendrite). High frequency stimulation induces LTP at pyramidal cell synapses that is NMDAR-independent and expressed presynaptically. Mechanisms involved are: increase in presynaptic calcium, adenylyl cyclase 1 (AC1), cAMP, protein kinase A (PKA), and ephrin signaling which up-regulate release machinery. At the presynaptic cite KARs and calcium release from stores might be involved, postsynaptic calcium increase due to the activation of mGluRs and VGCCs also might play a role. Same type of intense stimulation evokes LTD at filopodial synapses onto interneurons. At synapses expressing calcium-impermeable AMPARs (CI-AMPARs) LTD has a postsynaptic locus of induction and expression. It depends on calcium influx through NMDARs, and involves the endocytosis of surface AMPARs. LTD at synapses containing calcium-permeable AMPARs (CP-AMPARs) requires postsynaptic calcium increase, but is expressed presynaptically through activation of presynaptic mGluR7 and downstream Protein kinase C (PKC)-dependent cascades reducing neurotransmitter release probability. Low frequency stimulation evokes LTD at MF-pyramidal cell synapses. This form of LTD involves activation of presynaptic mGluR2, which in turn reduces cAMP level and subsequent decrease of PKA activity lowers the release probability.

It is widely accepted that MF LTP induction is independent of the activation of postsynaptic NMDARs (Harris and Cotman, 1986); reviewed by Nicoll and Schmitz (2005). In fact, evidence point to exclusive contribution of presynaptic mechanisms, including increase in presynaptic calcium currents and the activation of adenylyl cyclase-cAMP cascade, Figure 3 (Zalutsky and Nicoll, 1992; Weisskopf et al., 1994; Villacres et al., 1998). There are also data suggesting, that activation of presynaptic KARs and calcium release from internal stores might be involved (Contractor et al., 2001; Lauri et al., 2001, 2003; Bortolotto et al., 2003). However, several studies shed light on the importance of postsynaptic calcium signaling associated with VGCCs and mGluRs in MF LTP (Kapur et al., 1998, 2001; Yeckel et al., 1999; Wang et al., 2004). Pre and postsynaptic mechanism were suggested to be recruited based on the particular stimulus conditions used (Urban and Barrionuevo, 1996). While multiple evidence show that presynaptic enhancement of glutamate release is a key element in the development of MF LTP (Yamamoto et al., 1992; Zalutsky and Nicoll, 1992; Yeckel et al., 1999; Reid et al., 2004), postsynaptic regulation of MF LTP by retrograde ephrin signaling has also been proposed (Contractor et al., 2002; Armstrong et al., 2006).

Induction of LTD by low frequency stimulation is NMDAR independent, similarly to LTP (Kobayashi et al., 1996), and relies on the activation of presynaptic group II mGluRs, Figure 3 (Manzoni et al., 1995; Yoshino et al., 1996). Activation of mGluRs leads to decreased cAMP levels and subsequent PKA activation (Tzounopoulos et al., 1998), which in turn, down-regulates the vesicle release machinery and leads to synaptic depression. Thus, both LTD and LTP at MF-CA3 pyramidal cell synapses are expressed presynaptically and depend on cAMP production. LTD evoked with short depolarization (DiLTD) can only be observed at this synapse during the first 2–3 postnatal weeks. This form of plasticity is mediated by the transient presence of CP-AMPA receptors at MF-pyramidal cells synapse in these young animals (Ho et al., 2007).

This synapse also expresses a very unique form of plasticity, NMDARs mediated responses can be selectively potentiated, while the AMPAR component remains unaffected (Kwon and Castillo, 2008a; Rebola et al., 2008). This form of plasticity is mediated by the PKC-dependent recruitment of NMDARs. Interestingly, NMDAR-LTP participates in several metaplastic changes including: induction of NMDA-dependent LTP of AMPARs (Rebola et al., 2011) and a recently described heterosynaptic metaplasticity between MF and associational-commissural synapses (Hunt et al., 2013). Moreover, paired burst stimulation of presynaptic MFs and postsynaptic pyramidal cells, mimicking *in vivo* activity, efficiently induced bidirectional changes in the NMDAR-mediated currents. The direction of LTP was dependent on the timing of pre and postsynaptic bursts similarly to conventional spike-timing-dependent plasticity (Hunt et al., 2013). Bidirectional, long-term changes in NMDAR mediated responses provide higher degree of flexibility in information processing at this synapses.

### Long-term plasticity of at mossy fiber-interneuron synapses

Unlike in pyramidal cells, high frequency stimulation leads to LTD at synapses formed on interneurons (Maccaferri et al., 1998; Lei and Mcbain, 2004; Pelkey et al., 2005; Galvan et al., 2011). Furthermore, although this LTD could be equally evoked at synapses expressing calcium-permeable and calcium-impermeable AMPARs, both depending on postsynaptic calcium elevation, there is a drastic difference in the molecular mechanisms of LTD induction and expression at these two different types of synapses (Figure 3). At synapses expressing calcium impermeable AMPA receptors, LTD is expressed postsynaptically and it is NMDAR-dependent. Calcium entry through NMDAR triggers AP-2 dependent AMPAR internalization, by a mechanism similar to that at other synapses expressing NMDAR-dependent LTD (Lei and Mcbain, 2004). The fact that at this type of MF-interneuron synapse LTD seems to rely on a conventional molecular pathway is striking, given that this particular type of LTD could only be evoked with high and not low frequency stimulation which is commonly associated with LTD induction (Luthi et al., 1999; Lee et al., 2002).

At calcium-permeable AMPAR synapses LTD is NMDAR-independent, it requires calcium influx through AMPAR, but is expressed presynaptically via decreased glutamate release (Lei and Mcbain, 2004). The activation of mGluR7 which is selectively expressed on filopodia, but not the main MF bouton (Shigemoto et al., 1997), is required for this type of plasticity (Pelkey et al., 2005). This form of LTD involves the inhibition of calcium influx to the terminal through P/Q-type VGCCs (Pelkey et al., 2005). Since activation of presynaptic mGluR7s and postsynaptic calcium influx through AMPARs are necessary, this form of LTD most likely involves retrograde signaling, however its nature remains unknown (Pelkey et al., 2005). Interestingly, in synapses that contain a mixture of calcium-permeable and -impermeable AMPARs, LTD relies on presynaptic mechanisms (Lei and Mcbain, 2004).

Early studies did not reveal LTP at mossy fiber synapses formed on interneurons. However, subsequently it was discovered not only in dentate basket cells (Alle et al., 2001), but also in two types of CA3 inhibitory cells: stratum lucidum and stratum lacunosum-moleculare interneurons. Presynaptic form of LTP could be induced at calcium-permeable AMPAR containing MF synapses on stratum lucidum interneurons after prolonged application of mGluR7 agonist followed by high frequency stimulation. The initial LTD induced by the activation of mGluR7 was transformed into LTP after tetanus stimulation. This switch in the direction of plasticity occurred due to the internalization of surface mGluR7 receptors induced by L-(+)-2-Amino-4-phosphonobutyric acid (L-AP4) application (Pelkey et al., 2005). Receptor internalization is a key element of this phenomenon, as pharmacological blockade of mGluRs was not sufficient to unmask LTP at this synapse. Internalization of mGluR7 changes the direction of plasticity by turning on release sensitivity to cAMP. At naive mGluR7 expressing synapses cAMP elevation does not have any effect, while at synapses with internalized mGluR7 it leads to strong potentiation (Pelkey et al., 2008). Moreover, the molecular cascaded involves adenylate cyclase and PKA activation, as well as an active zone protein replication in mitochondria 1a (RIM1a). Therefore, this synapses shows bidirectional LTP, dependent on the presence of mGluR7, which adds an additional layer of complexity to the interaction between different forms of plasticity at the same synapse.

In stratum lacunosum-moleculare interneurons classic, high frequency stimulation of MFs triggered LTP. This form of LTP is expressed postsynaptically at synapses containing calcium-impermeable AMPARs, but in contrast to the plasticity at calcium-impermeable AMPAR synapses on stratum lucidum interneurons, it does not depend on the activation of NMDARs (Galvan et al., 2008). It involves activation of mGluR1 and could be mimicked by either application of forskolin, indicating the involvement of the cAMP/PKA signaling cascade, or by PKC activation. Downstream of mGluR1 activation, postsynaptic calcium signaling, including calcium release from internal stores and influx through L-type VGCCs is necessary for this form of plasticity. When calcium signaling is blocked, the direction of plasticity is reversed and high frequency stimulation leads to LTD (Galvan et al., 2008).

## Conclusion

MF projection to the CA3 area is a remarkably complex system both anatomically and functionally. Synaptic communication between MFs and their target cells is regulated in an activity- and target cell-dependent manner. We are only at the beginning of understanding the mechanism by which these diverse synaptic responses combine to determine how dentate granule cells translate cortical information to hippocampal code. While intricate details of synaptic interaction between granule cells and their postsynaptic targets are well known, the major outstanding question is how *in vivo* activity patterns of several presynaptic cells combine at single cell and population level. In order to properly investigate this question, simultaneous detection of *in vivo* activity patterns of pre and postsynaptic cells and their exact connectivity patterns needs to be defined. Recent advances in imaging techniques using voltage-sensitive and calcium dyes and viral technologies allowing the visualization of certain subset of neurons and their projections could help to attain this aim.

## Conflict of interest statement

The authors declare that the research was conducted in the absence of any commercial or financial relationships that could be construed as a potential conflict of interest.
